# Supplementary material for: The Apelin receptor enhances Nodal/TGFβ signaling to ensure proper cardiac development
Source: eLife. 2016 Apr 14;5:e13758. doi: 10.7554/eLife.13758 (PMC4859801; doi:10.7554/eLife.13758)
Supplement: Figure 4—source data 1. — Fold change for all mesp family probes present on the microarray. Fold change is comparing WT to aplnra/b morphant embryos from normalized data from four biological replicates. DOI: http://dx.doi.org/10.7554/eLife.13758.011 [file elife-13758-fig4-data1.docx]

| **Probe ID** | **Gene Name** | **Fold Change** |
| --- | --- | --- |
| A_15_P116279 | *mespaa* | -3.24 |
| A_15_P627536 | *mespaa* | -3.04 |
| A_15_P630646 | *mespba* | -2.08 |
| A_15_P108949 | *mespba* | -2.04 |
